# Fetoplacental vascular effects of maternal adrenergic antihypertensive and cardioprotective medications in pregnancy

**DOI:** 10.1097/HJH.0000000000003532

**Published:** 2023-09-11

**Authors:** Teresa Tropea, Weerawaroon Mavichak, Angelos Evangelinos, Charlotte Brennan-Richardson, Elizabeth C. Cottrell, Jenny E. Myers, Edward D. Johnstone, Paul Brownbill

**Affiliations:** a Maternal & Fetal Health Research Centre, Division of Developmental Biology & Medicine, School of Medical Sciences, Faculty of Biology, Medicine and Health, University of Manchester; b St Mary's Hospital, Manchester University Hospital NHS Foundation Trust, Manchester Academic Health Science Centre, Manchester, UK

**Keywords:** adrenergic antihypertensive/cardioprotective medications, adrenergic receptors, endothelial/smooth muscle cells, fetoplacental vasculature, pregnancy

## Abstract

Maternal cardiovascular diseases, including hypertension and cardiac conditions, are associated with poor fetal outcomes. A range of adrenergic antihypertensive and cardioprotective medications are often prescribed to pregnant women to reduce major maternal complications during pregnancy. Although these treatments are not considered teratogenic, they may have detrimental effects on fetal growth and development, as they cross the fetoplacental barrier, and may contribute to placental vascular dysregulation. Medication risk assessment sheets do not include specific advice to clinicians and women regarding the safety of these therapies for use in pregnancy and the potential off-target effects of adrenergic medications on fetal growth have not been rigorously conducted. Little is known of their effects on the fetoplacental vasculature. There is also a dearth of knowledge on adrenergic receptor activation and signalling within the endothelium and vascular smooth muscle cells of the human placenta, a vital organ in the maintenance of adequate blood flow to satisfy fetal growth and development. The fetoplacental circulation, absent of sympathetic innervation, and unique in its reliance on endocrine, paracrine and autocrine influence in the regulation of vascular tone, appears vulnerable to dysregulation by adrenergic antihypertensive and cardioprotective medications compared with the adult peripheral circulation. This semi-systematic review focuses on fetoplacental vascular expression of adrenergic receptors, associated cell signalling mechanisms and predictive consequences of receptor activation/deactivation by antihypertensive and cardioprotective medications.

## INTRODUCTION

Maternal cardiovascular disease affects up to 4% of pregnancies [1]. In women with cardiac conditions, a failure of the maternal haemodynamic adaption to pregnancy may have detrimental effects, increasing the risk of morbidity and mortality for both the mother and the fetus [2]. In up to 10% of pregnancies, maternal blood pressure exceeds 140 mmHg systole and/or 90 mmHg diastole [3], which is a significant risk factor for fetal growth restriction (FGR), preterm delivery and adverse perinatal outcomes [3].

Normal pregnancy is sustained by a progressive physiological adaptation of the maternal cardiovascular system across gestation. These changes include an increase in maternal cardiac output up to 50%, and a reduction in both the mean arterial pressure and the systemic vascular resistance [4] for adequate oxygen and nutrient delivery to the growing fetus. Dramatic renal haemodynamic changes contribute significantly to increase maternal plasma volume. From early stages, pregnancy increases renal blood flow up to 80% and glomerular filtration rate to 50% compared with the nonpregnant state [5]. However, in normal pregnancy, most women increase their antidiuretic hormone levels to prevent polyuria [6].

Pregnant women with hypertension and cardiac conditions are often prescribed adrenergic medications, mainly β-adrenergic receptor antagonists and α-adrenergic receptor agonists, to manage maternal disease [7]. Although these treatments may confer improvements in maternal cardiovascular function, these medications may potentially compromise fetal growth and development [7–9] likely due to differences in adrenergic receptor expression and undesirable effects on the fetoplacental circulation.

Placental blood flow increases as pregnancy progresses to satisfy the requirements of oxygen and nutrients for normal fetal growth and development. Functional regulation of fetoplacental vascular tone, mainly mediated by the action of paracrine and autocrine vasodilators (i.e. nitric oxide, NO; prostacyclin, PGI2; hydrogen sulphide, H2S) and vasoconstrictors (i.e. endothelin-1, ET-1; thromboxane, TXA_2_), contributes to achieve optimal placental perfusion throughout pregnancy [10]. Ultrasound is an important tool in obstetric medicine to assess fetal growth and blood flow between the placenta and fetus; growth scans and umbilical artery Doppler ultrasound velocimetry indices are used to provide an evaluation of fetoplacental blood flow resistance to detect complications. In cases of FGR, umbilical artery Doppler is often used to inform timing of birth decisions given the increased risk of stillbirth associated with abnormal Doppler indices [11].

Pregnancies complicated by maternal hypertension and cardiac conditions are associated with reduced fetoplacental blood, suggesting fetoplacental malperfusion [12–14]. Clinical trials and studies in animal and ex-vivo models have shown the likely association between poor fetal outcomes (such as fetal growth restriction, FGR, preterm birth) and the potential effect of adrenergic medications to reduce placental blood flow and perfusion [15–26].

To the best of our knowledge, there is no conclusive evidence which addresses the mechanisms by which adrenergic medications may affect fetoplacental vascular tone through activation/deactivation of specific adrenergic receptors and the inherent signalling pathways that may contribute to FGR. This semi-systematic review aims to tackle this evidence gap by summarizing gene/protein expression of adrenergic receptors, mechanistic signalling elicited by adrenergic receptors and effects evoked by adrenergic medications through activation/deactivation of adrenergic receptors on the fetoplacental vasculature. We focus here on data from umbilical vessels, chorionic plate vessels and placental villous tree as component parts of the fetoplacental vasculature involved in the overall control of placental vascular resistance [10].

## MATERIALS AND METHODS

### Research strategy

**FIGURE 1:**
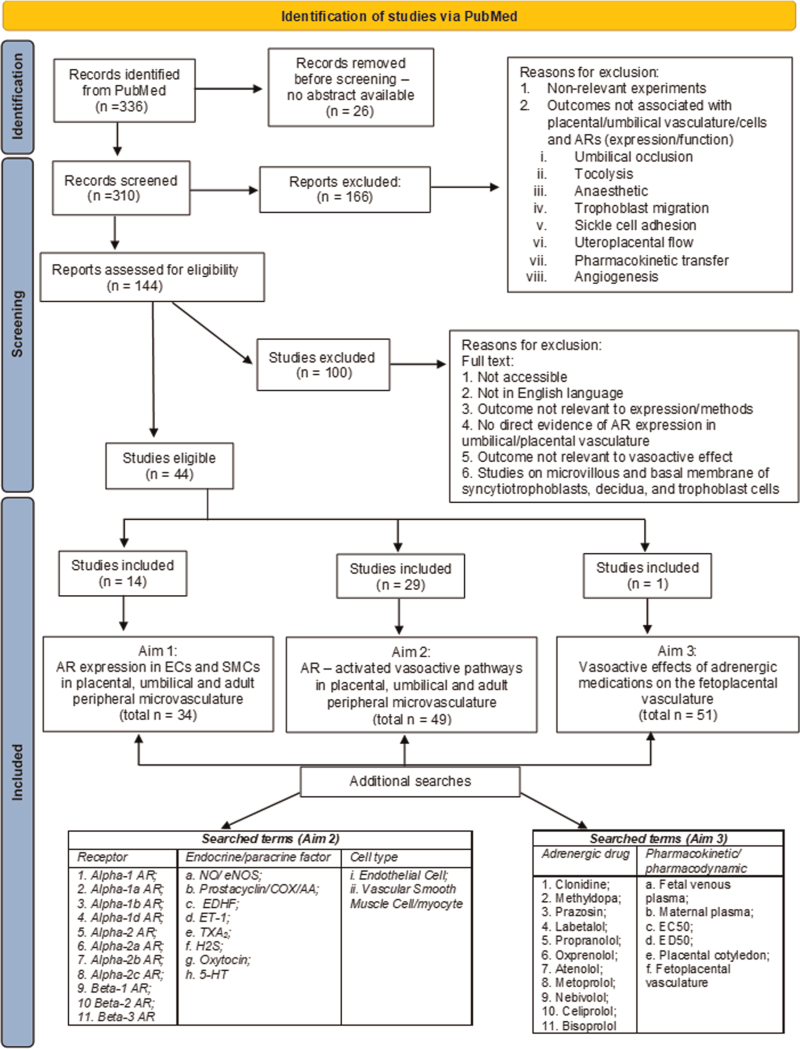
PRISMA flow diagram of searches conducted in PubMed. One hundred and thirty-four articles were included in the final analysis. Additional searches were performed to complete Aim 3 and, where relevant, were included in Aim 1 and 2. 5-HT, serotonin; AA, arachidonic acid; AR, adrenergic receptor; COX, cyclooxygenase; EC50, half-maximal effective concentration; ED50, effective dose for 50% of the population; EDHF, endothelium-derived hyperpolarizing factor; eNOS, endothelial nitric oxide synthase; ET-1, endothelin-1; H2S, hydrogen sulphide; NO, nitric oxide; TXA2, thromboxane A2.

To explore the complexity of potential effects orchestrated by the action of adrenergic antihypertensive and cardioprotective therapies on adrenergic receptors within the fetoplacental vasculature, a semi-systematic literature search was initially conducted in PubMed using the following terms: ‘adrenergic receptor (placenta OR umbilical)’, which yielded 336 records. Peer-reviewed articles were screened from titles and abstracts with no discrimination against the year of publication, methodologies, health status of placentas or human and animal species. Only studies focussing on the expression and/or on the function of adrenergic receptors in placental, umbilical and adult peripheral microvasculature were included in Aim1 and Aim3, respectively. Studies on adrenergic receptors but focusing on processes not directly mediating vasoactive pathways (e.g. angiogenesis) were excluded. As the fetal side of the placenta is unique, as it is not innervated [27], studies on uteroplacental blood flow were also excluded (Fig. 1, PRISMA flow diagram).

To provide detailed downstream signalling pathways, additional studies were found in Google Scholar using a combination of keywords (Fig. 1) that identified full-text, peer-reviewed studies related to adrenergic receptors in the umbilical and placental vasculature, and the placental microvasculature (e.g. alpha-1 adrenergic receptor and nitric oxide and endothelial cell).

To predict potential effects of antihypertensives on the targeted adrenergic receptors on the fetoplacental vasculature, Aim 3 required additional searches performed on Google Scholar and PubMed. These studies delineate the adrenergic antihypertensives’ profiles and pharmacological parameters, and if related to adrenergic receptor expression and/or function, were included in Aim 1 and 2, respectively. Only peer-reviewed articles arising from all potential combination of keywords listed in Fig. 1 were included (e.g. clonidine and foetal venous plasma; clonidine and EC50).

### Adrenergic receptors and signalling in cardiomyocytes, endothelial cells and smooth muscle cells

A maternal cardiovascular system with low peripheral resistance, increased cardiac output and reduced maternal blood pressure is crucial for successful fetoplacental development. The adrenergic system contributes significantly to the maternal cardiovascular adaptations of pregnancy by regulating uteroplacental vascular tone and blood pressure [28]. Adrenergic receptors belong to the superfamily of G protein-coupled receptors (GPCRs), which, depending on specific ligand-binding, can stimulate or inhibit adenylyl-cyclase (AC), via activation of Gs and Gi proteins, respectively, and activate phospholipase C (PLC) by modulating Gq proteins [29,30]. Adrenergic receptors include α and β-receptors. α-ARs are divided into α_1_ (α_1A_-AR, α_1B_-AR, α_1D_-AR) and α_2_ (α_2A_-AR, α_2B_-AR, α_2C_-AR) subtypes; β-ARs include β_1_, β_2_ and β_3_ subtypes [29,30]. It is still controversial whether α_1L_ and β_4_ subtypes are additional subtypes or conformational states of α_1_-AR and β_1_-ARs, respectively [30]. Specifically, vasoconstriction and vasorelaxation promoted by α1-AR and α2-ARs, respectively, are increased, whereas vasorelaxant responses to β-AR activation are reduced *ex vivo* in uterine arteries of pregnant rats [31]. A general description of the adrenergic receptors in cardiomyocytes, endothelial cells and vascular smooth muscle cells (VSMCs) is reported in Table 1[32–54].

**TABLE 1:** General overview of adrenergic receptors in cardiomyocytes, endothelial and vascular smooth muscle cells

Cell type	Receptor	Signalling	Effect	References
Cardiomyocytes	α_1A_, α_1B_ (20% of ARs)	Gq / PLC-β / PKC / IP_3_ / increased Ca^2+^	Myocardial contractility (positive inotropy)	[32,33]
	α_2_	Gi / NO release / decreased Ca^2+^	Myocardial relaxation (positive lusitropy)	[34,35]
	β_1_ (mainly), β_2_	Gs / AC / increased cAMP / activation of PKA / phosphorylation of phospholamban, troponin I, sarcoplasmic reticulum–located ryanodine receptors and L-type Ca^2+^ channels	Increased heart rate (positive chronotropy), positive inotropy and positive lusitropy	[36]
	β_3_ (low expression)	Gs and/or Gi	Stimulatory and/or inhibitory effect	
Endothelial cells	α_1_ (α_1D_ mainly)	Gq / PLC/ NO release	Vasorelaxation (animal pulmonary, brachial, mesenteric arteries and coronary microvessels)	[37–40]
	α_2_ (α_2A/D_ mainly)	PGI2 and NO release	Vasorelaxation (animal aortic rings, bronchial and epicardial coronary arteries)	[37,41,42]
	β (β_2_ and β_3_ mainly)	PGI2, NO and EDHF release	Vasorelaxation (HUVECs, human coronary microarteries, animal thoracic aorta)	[43–46]
Vascular smooth muscle cells	α_1_ (α_1A_ and α_1D_ mainly)	Gq / PLC-β / PKC / IP3 / increased Ca^2+^ ?	Vasoconstriction (animal mesenteric artery and thoracic aorta)	[47–49]
	α_2_ (α_2A_ and α_2C_ mainly)	increased Ca^2+^ ?	Vasoconstriction (animal bronchial artery) Regulation of vascular tone (humans)	[30,37,41]
	β (β_1_^a^ and β_2_ mainly)	increased cAMP and K+ channel activation	Vasorelaxation (animal aortic ring, mesenteric artery)	[30,50–54]

AC, adenylyl cyclase; cAMP, cyclic adenosine monophosphate; EDHF, endothelium-derived hyperpolarizing factor; HUVECs, human umbilical vein endothelial cells; IP_3_, inositol (1,4,5)trisphosphate; NO, nitric oxide; PGI2, prostacyclin; PKA, protein kinase A; PKC, protein kinase C; PLC-β, phospholipase C-β.

a β_1_ represents the main subtype in epicardial coronary and middle cerebral arteries in both human and animal VSMCs.

**FIGURE 2:**
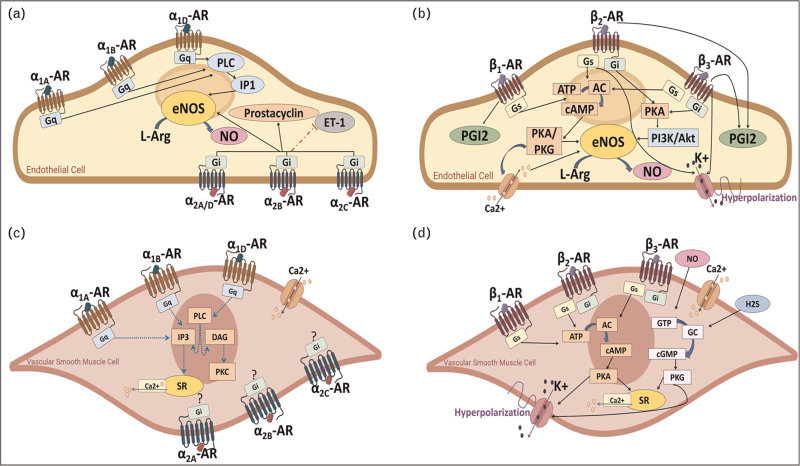
Major vasoactive pathways activated by adrenergic receptors in the systemic vasculature. Black solid arrows indicate intracellular pathways eliciting vasorelaxation promoted by activation of α-ARs (a) and β-ARs (b) in endothelial cells, and by β-ARs in vascular smooth muscle cells (d). Blue dotted arrows indicate intracellular pathways causing vasoconstriction promoted by α-ARs in vascular smooth muscle cells (c). Red dash dotted line indicates inhibition of ET-1 production (a). Green solid arrows indicate calcium release from the sarcoplasmic reticulum (SR; c and d).

Overstimulation of the adrenergic system may compromise uteroplacental perfusion [28]; however, it is essential that maternal cardiac output is sufficiently increased to sustain fetal growth through the uteroplacental circulation. Careful considerations have to be accounted for when prescribing adrenergic medications restoring cardiac function, especially β_1_-AR blockers, in pregnancy. The potency of agonists-binding receptors on both the endothelium and the smooth muscle layer, in combination with signalling effects, determines the overall response of a vessel in terms of changes in vascular tone. The major vasoactive pathways activated by α-AR and β-ARs in endothelial cells and VSMCs are summarized in Fig. 2. With the exception of HUVECs, these findings arise from studies conducted in the systemic vasculature.

### Gaps in knowledge in pregnancy

**FIGURE 3:**
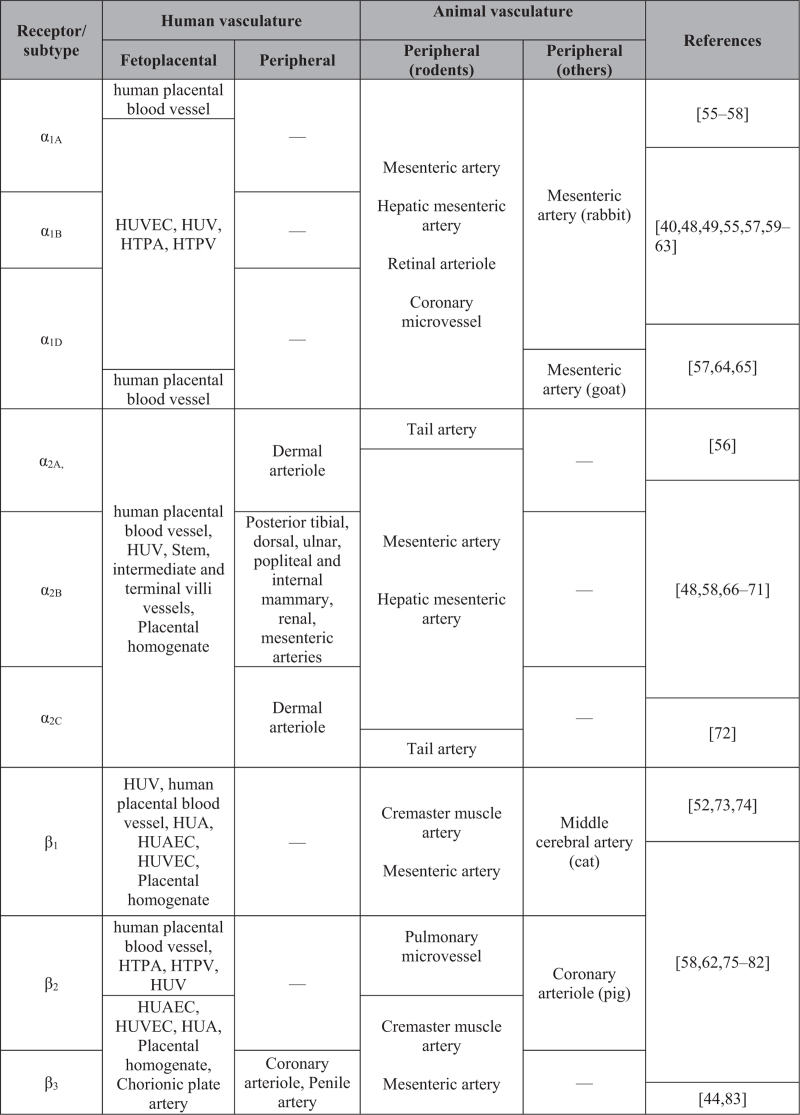
Relative AR expression in the fetoplacental vs. adult peripheral microvasculature. HTPA, human term placental artery; HTPV, human term placental vein; HUA, human umbilical artery; HUAEC, human umbilical artery endothelial cell; HUV, human umbilical vein; HUVEC, human umbilical vein endothelial cell. Vasculature and reference columns are not spaced according to receptor subtype, but according to commonalities.

The absence of neuronal control in the placenta [27] does not exclude adrenergic signalling within the fetoplacental vasculature, as circulating catecholamines still act upon the receptors that are present. As we summarized in Aim 1, previous studies consistently show evidence of adrenergic receptor expression within the fetoplacental vasculature (Fig. 3), suggesting that agonist-activated adrenergic receptor pathways may alter vascular tone in the fetoplacental circulation [40,44,48,49,52,55–83].

Despite the extensive use of adrenergic medications in pregnant women, including β-blockers as first-line treatments [84], data supporting their safety on the fetus are limited. Whether all adrenergic receptor subtypes are expressed in resistance-size placental arteries, such as in the chorionic plate arteries, and how adrenergic receptor paracrine signalling between endothelial cells and VSMCs may affect fetoplacental vascular tone and blood flow supply to the fetus is currently unknown. Importantly, there is no clear evidence of the consequences of adrenergic receptor blockade on the maintenance of blood flow through the fetoplacental circulation and whether the likely vascular action mediated by adrenergic medications is associated with adrenergic receptor affinity, gestational age, dose-regimen and administration route of the drugs. Most of these therapies are prescribed to pregnant women on a relative risk-basis. However, the pharmacokinetics of the drugs may dramatically change in pregnancy compared with the nonpregnant state [85]. Furthermore, there is a paucity of data with regards to fetal plasma concentrations and the likely evoked effect of the medication on fetoplacental vascular tone. We acknowledge that the few functional data available are obtained by ex-vivo experimentation, without accounting for by-products of drug metabolism *in vivo*. To address all unknowns related to the implications of in-utero administration of adrenergic receptor antihypertensives, with regards to the regulation of vascular tone, a comprehensive understanding of the expression/function of adrenergic receptors in the fetoplacental vasculature is paramount. This information could provide an evidence base to guide clinicians on the choice of the safest option for fetal outcomes, concurrently with the most effective adrenergic medication for women with cardiovascular disorders in pregnancy.

### Aim 1. Adrenergic receptor expression in the fetoplacental and in the adult peripheral microvasculature

In adults, the peripheral microcirculation has the crucial function of regulating nutrients/waste exchange and controlling local vascular tone and blood perfusion within the tissues [86]. Similarly, the fetoplacental vasculature mediates a fine control of blood flow by maintaining low resistance and high flow throughout gestation, to deliver sufficient oxygen and nutrients to the fetoplacental circulation for normal fetal growth [87]. Although sympathetic nervous system stimulation is known to regulate vascular tone in the adult peripheral circulation [30], the fetoplacental circulation is devoid of such innervation [27]. Although adrenergic receptors are widely distributed within the cardiovascular system, with locus-specific and functional expression depending on both animal species and the vascular bed [30], limited information is available with respect to the human fetoplacental microcirculation. Figure 3 summarizes current information on relative adrenergic receptor expression in the fetoplacental and adult peripheral microvasculature of humans, rodents and other species (goat, pig, cat and rabbit).

#### Expression of adrenergic receptor subtypes in the fetoplacental circulation

As summarized in Figure 3, there is evidence showing that all adrenergic receptor subtypes are expressed in the human fetoplacental vasculature. The majority of studies have been conducted using HUVECs or whole placental homogenates, and reported the expression of α_1A_, α_1B_, α_1D_, α_2A_, α_2B_, α_2C_[58,63,69,70] and β_1_, β_2_ and β_3_ adrenergic receptor subtypes [58,73,74,76,79], without clarifying the locus or the vessel type [70,73,74,76] and with a likely high background expression from trophoblast cells [69]. α_2A_ appears to be the only adrenergic receptor subtype identified in both endothelial cells and VSMCs within the blood vessels of stem, intermediate and terminal villi [69]. Only one study has shown the expression of β_2_-AR and β_3_-ARs in chorionic plate arteries, although with no distinction between the endothelial and the smooth muscle cell layer [80]. Most noninnervated human vascular expression studies relate to the umbilical vein and not the placental microvasculature (α_1A_, α_1B_ and α_1D_ subtypes) [55,58]; (α_2A_, α_2B_, and α_2C_-ARs and β_1_ and β_2_ subtypes) [58,75] (Fig. 3). The same is true for β-ARs, with much evidence coming from the umbilical vessels: human term placental vein (HTPV) expresses β_2_ subtypes [62]. In this same study, the authors did not investigate β_1_-AR and β_3_-ARs, but showed expression of α_1A_, α_1B_, α_1D_ subtypes in human term placental artery (HTPA) [62]. All three β-AR subtypes are expressed in human umbilical artery (HUA) [75,79], whereas only β_2_-AR has been shown in HTPA [62]. However, the methodologies used in these studies clearly represent a limitation for a detailed localization of adrenergic receptor expression sites in the fetoplacental microvasculature, and adrenergic receptor expression patterns in this unique noninnervated circulation remain poorly defined in the literature. Further localization of adrenergic receptor subtypes’ expression is needed before the contribution of these receptors to vasoactive function and the targeted effects of adrenergic medications on endo-myocyte signalling in the fetoplacental microvasculature can be fully understood.

### Aim 2. Adrenergic effects in blood vessel: component parts of physiological mechanisms

Although not innervated, the expression of extra-neuronal monoamine and norepinephrine transporters in trophoblast, stroma and myofibroblasts associated with fetoplacental vessels, confers the placenta a likely protective role in clearing endogenous catecholamines from the fetal and maternal circulations [88]. Some evidence has suggested that in hypertensive pregnancies, these transporters are downregulated, thus increased levels of endogenous catecholamines may compromise this placental defence [89]. In the plasma of preterm newborns, the concentrations of both adrenaline and noradrenaline are approximately two-fold higher compared with full-term babies [90]. Adrenaline levels have been found to be four-fold higher in preterm new-borns with respiratory distress syndrome, and eight-fold higher in those with placental insufficiency [90]. A study investigating fetal plasma noradrenaline responses to invasive procedures showed no correlation between maternal and fetal plasma levels of noradrenaline [91]; furthermore, although physiological infusion of adrenaline in pregnant sheep elevated fetal plasma adrenaline, the reduction of uterine, but not umbilical blood flow [92], suggests the likely barrier function of the placenta. In addition, ex-vivo perfusion studies using isolated human placental cotyledons have shown no change in fetal arterial perfusion pressure following exposure to adrenaline and noradrenaline via the maternal circulation [93]. This suggests that either adrenaline does not transfer across the placenta, or there is a different response within the fetoplacental circulation.

Changes in the release of the endogenous catecholamines from the sympathetic nervous system regulate vascular tone through the activation of adrenergic receptors [30]. Endothelial factors, including nitric oxide, PGI2, H2S and EDHF, are well known to evoke vasorelaxation, whereas the production of ET-1, TXA2 or VSMC depolarization causes vasoconstriction [10]. Figure 4  summarizes the major mechanisms initiated by the activation of adrenergic receptors in the endothelium and in the VSMC layer of resistance-size vessels in animal models, and in the human fetoplacental vasculature [94–131].

**FIGURE 4:**
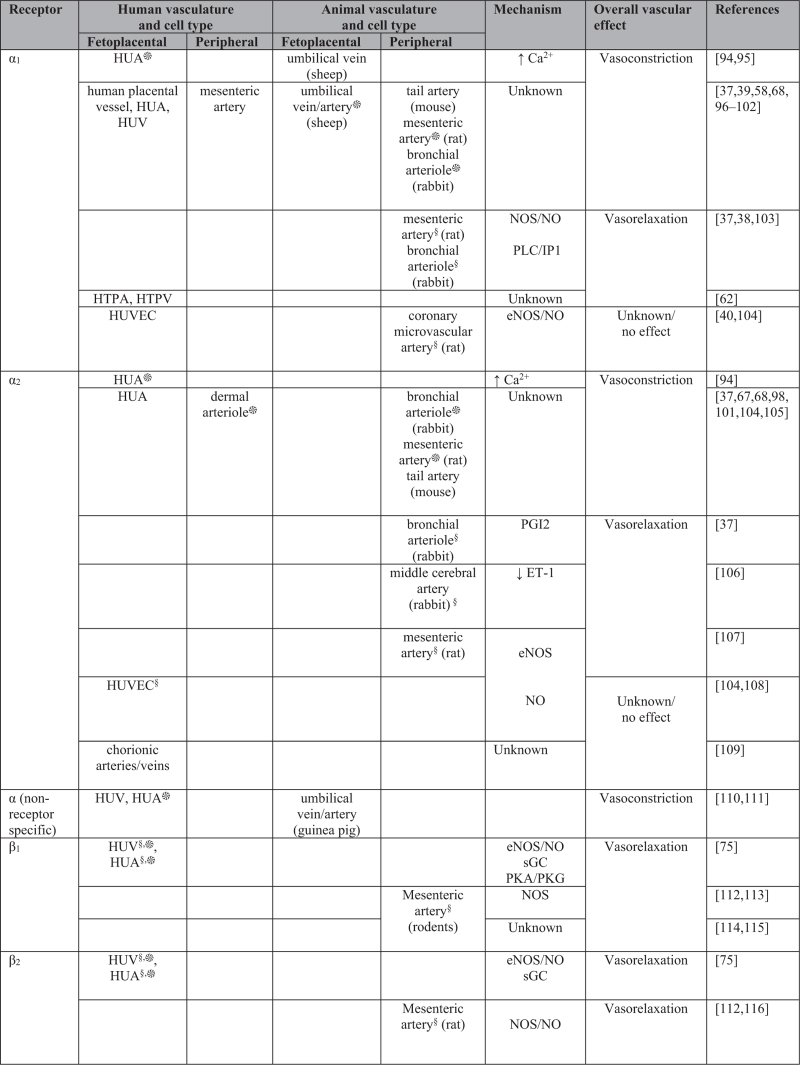
Mechanisms underlying adrenergic receptor mediated vasoactive effects in the fetoplacental and in the adult peripheral microvasculature. Akt, protein kinase B; Ca^2+^, calcium; cAMP, cyclic adenosine monophosphate; eNOS, endothelial nitric oxide synthase; ET-1, endothelin-1; HUA, human umbilical artery; HTPA, human term placental artery; HTPV, human term placental vein; HUV, human umbilical vein; HUVEC, human umbilical vein endothelial cells; H2S, hydrogen sulphide; IP1, inositol monophosphate; NO, nitric oxide; NOS, nitric oxide synthase; PGI2, prostacyclin; PI3K, phosphoinositide 3-kinase; PKA, protein kinase A; PKG, protein kinase G; PLA2, phospholipase A2; PLC, phospholipase C. Cell type: ^§^endothelial cell; ^∗^vascular smooth muscle cell.

**FIGURE 4 (Continued):**
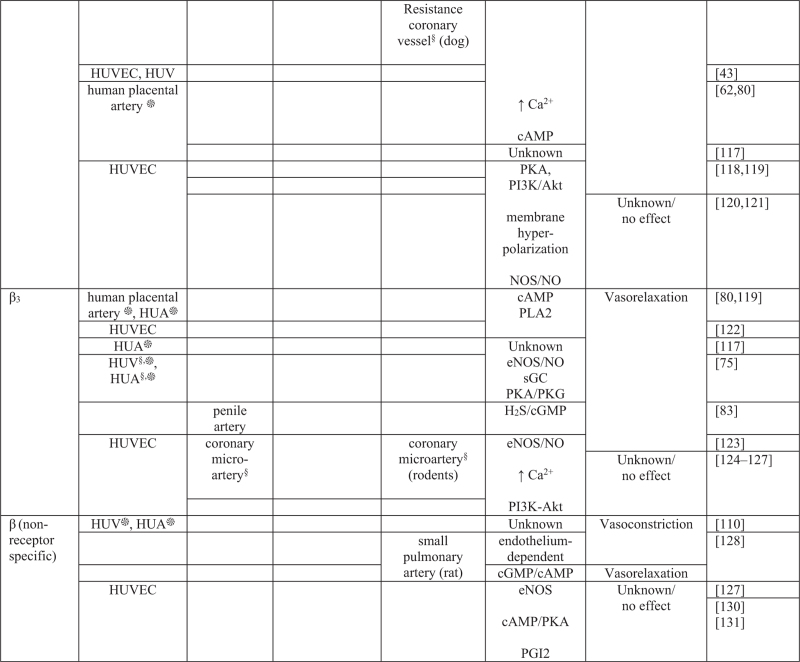
Mechanisms underlying adrenergic receptor mediated vasoactive effects in the fetoplacental and in the adult peripheral microvasculature. Akt, protein kinase B; Ca^2+^, calcium; cAMP, cyclic adenosine monophosphate; eNOS, endothelial nitric oxide synthase; ET-1, endothelin-1; HUA, human umbilical artery; HTPA, human term placental artery; HTPV, human term placental vein; HUV, human umbilical vein; HUVEC, human umbilical vein endothelial cells; H2S, hydrogen sulphide; IP1, inositol monophosphate; NO, nitric oxide; NOS, nitric oxide synthase; PGI2, prostacyclin; PI3K, phosphoinositide 3-kinase; PKA, protein kinase A; PKG, protein kinase G; PLA2, phospholipase A2; PLC, phospholipase C. Cell type: ^§^endothelial cell; ^∗^vascular smooth muscle cell.

A number of studies on both the fetoplacental and the peripheral vasculature have shown that activation of α-ARs on VSMCs induces vasoconstriction, whereas activation of α-ARs on endothelial cells promote vasorelaxation mainly through eNOS activation/nitric oxide and PGI2 release (Figs. 2 and 4 ). Findings specific for α-ARs reported that activation of α_1A_ and α_1D_ subtypes releases nitric oxide in rat coronary microvessels [40], and α_1D_-ARs promotes vasorelaxation, whereas α_1A_-ARs lead to vasoconstriction in rat mesenteric arteries [39]. Studies in human fetoplacental blood vessels demonstrated that α_1A_, α_1B_ and α_1D_ subtypes promote vasoconstriction [62,96]. Amongst α_2_ subtypes, α_2D_ exerts a vasorelaxant effect by producing nitric oxide in rat mesenteric arteries [107], whereas α_2A_ and α_2C_ evoke vasoconstriction in human dermal arteriole [67] and in mouse tail artery [68]. In contrast, and with few exceptions, agonist-activation of β-AR subtypes on either the endothelium or on VSMCs evokes vasorelaxation. This vasodilatory effect has been associated to several pathways, including eNOS/NO signalling and H2S production in endothelial cells, endothelium-independent mechanisms and activation of PKA/PKG, cGMP/cAMP pathways in SMCs (Figs. 2 and 4 ).

### Aim 3. Prediction of effect of adrenergic medications on fetoplacental vascular tone

#### Antihypertensive and cardioprotective medications prescribed in pregnancy

Adrenergic therapies are prescribed in pregnancy to improve maternal cardiovascular function. Amongst these medications, β-AR antagonists are the most commonly prescribed to reduce maternal blood pressure and/or heart rate in the context of impaired left ventricular function (Table 2) [132,136–143]. These drugs can cross the placental barrier and it is possible they have a detrimental effect on placental vascular function and fetal growth and development. Chronic exposure to β-AR antagonists has been shown to increase the risk of fetal hypoglycaemia, bradycardia [133] and cardiovascular defects in the newborn [134], and has been associated with an increased risk of FGR [135]. However, it is unknown whether these effects are mediated through an impaired fetoplacental vascular function or are attributable to the underlying maternal disease.

**TABLE 2:** Major adrenergic medications prescribed in pregnancy

Adrenergic medications	Agonist/Antagonist
Clonidine	α_2_-AR – agonist [136]
Methyldopa	α_2_-AR – agonist [137]
Prazosin	competitive α_1_-AR – antagonist [23]
Labetalol	β-AR and α_1_-AR antagonist, partial β_2_-AR agonist [138]
Propanolol	β-AR antagonist [139]
Oxprenolol	β-AR antagonist, partial β-AR agonist [140]
Atenolol	β_1_-AR antagonist, low affinity β_2_- and β_3_-AR antagonist [141]
Bisoprolol	β_1_-AR antagonist [20]
Metoprolol	β_1_-AR antagonist [15]
Nebivolol	β_1_-AR antagonist, weak β_2_-AR antagonist, β_3_-AR agonist [142]
Celiprolol	β_1_-AR and weak α_2_-AR antagonist, β_2_-AR agonist [143]

Clinical studies have reported an association between maternal administration of labetalol, propranolol, metoprolol or atenolol and low birthweight [15,144]; in addition, atenolol has been demonstrated to reduce fetal heart rate and umbilical blood flow, and is no longer recommended for use in pregnancy [145]. Contrasting evidence, based mainly on the lack of adverse effects on uteroplacental blood flow, have shown that labetalol, methyldopa, metoprolol and propranolol are well tolerated for the fetus [15,146–148]. In contrast, an observational cohort study reported reduced birthweight in pregnancies exposed to bisoprolol during first trimester [20]. Similarly, treatment with either nebivolol or bisoprolol reduced uteroplacental blood flow and caused FGR in pregnant rats [21]. FGR has also been reported in pregnant women medicated with prazosin, but there is no evidence of its effect on uteroplacental blood flow [22]. One of the major limitations of these clinical studies is the lack of data related to placental vascular resistance. Interesting results have been shown in ex-vivo studies testing the first-line antihypertensives used in the UK, labetalol and methyldopa. Labetalol is a competitive and nonselective α_1_-AR and β-AR antagonist, with associated intrinsic sympathomimetic activity on β_2_-AR [149]. One study using ex-vivo dual perfusion of the human placental cotyledon demonstrated that labetalol potentiates the thromboxane vasoconstriction response, at a concentration of 10^–6^ mol/l [24]. It is worth nothing that, compared with the levels of labetalol found in the fetal plasma, the concentration of labetalol used in the ex-vivo study was nearly two orders of magnitude higher [150,151]. In contrast, a study performed in human umbilical arteries has shown that labetalol causes vasorelaxation, whereas methyldopa evokes vasoconstriction in the same vascular bed [105]. Therefore, it is vital to investigate the mechanisms elicited by adrenergic medications on the fetoplacental vasculature to delineate the relative consequences in terms of changes in vascular tone on fetal growth.

#### Likely net vasoactive effect

The effect of adrenergic medications on the fetoplacental vasculature, with regard to the regulation of vascular tone, is unknown. One key aim of this review was to predict the likely vasoactive effects mediated by adrenergic medications on the fetoplacental vasculature, by accounting for the intrinsic response of the drugs binding to the adrenergic receptors expressed on the systemic vasculature, and their plasma levels. To interpret the pharmacokinetic data for prediction of pharmacodynamic effects, we included information on the drug levels found in the fetal plasma or, in the absence of this evidence, we accounted for the fetal:maternal (F:M) plasma level ratios. The pharmacokinetic and pharmacodynamic parameters of the most commonly prescribed adrenergic medications are summarized in Supplementary Table 1. Prediction of potential vasoactive effects mediated by adrenergic medications on the fetoplacental vasculature (Supplementary Table 1) is mainly based on nonfetoplacental and animal studies. Except for labetalol and propranolol, there are currently insufficient human data to predict the likely net vasoactive effect of adrenergic medications on fetoplacental vascular tone. Importantly, data collected from nonpregnant individuals suggest that levels of labetalol may exceed 10^−6^ mol/l when the drug is administered intravenously [152]. As such, a potential intravenous administration of labetalol in pregnant women may increase peak fetal venous plasma to a concentration, which is likely to increase fetoplacental vascular tone based on ex-vivo studies [24] (Supplementary Table 1). On the contrary, oral administration of labetalol is unable to cause any vasoactive effect on the fetoplacental vasculature, as fetal plasma levels of labetalol do not reach 10^−6^ mol/l [150,151]. The nonselective β-AR antagonist, propranolol, has been shown to elicit a slight vasorelaxant effect (∼ 16%) on human placental artery and vein [153]. The low magnitude of the change in vascular tone has been associated with the lack of innervation in the fetoplacental vascular bed [153]. We speculate that the blockade action of propranolol on β-ARs may not promote any change in the fetoplacental vascular tone, based on evidence of dosage. High half maximal inhibitory/effective values vs. several orders of magnitude lower concentrations of adrenergic medications found in the fetal plasma, led us to speculate that these drugs are unlikely to elicit either vasoconstriction or vasodilation in the fetoplacental vasculature (Supplementary Table 1), but again, direct evidence is lacking.

The maternal plasma albumin concentration decrease by approximately 14% across gestations has been attributed to an increase in renal clearance and an increase in maternal plasma volume [154,155]. However, evidence that the maternal alpha-1 acid glycoprotein level does not change across gestation suggests that this is predominantly due to changes in the synthesis catabolism rates of albumin [156]. Labetalol and propanolol have been reported to bind nearly 50% to albumin [157,158]. A reduction in the concentration of albumin might therefore have a small impact on transplacental transfer pharmacokinetics of albumin-bound drugs, but pharmacokinetics is complex and other factors may play a more important role. This is evidenced in the transfer of rosiglitazone and glyburide to the fetal circulation; both have an equal and very high binding capacity for albumin, but with very different transfer efficiencies; efflux transporter activity of the placental barrier and the lipophilic nature of the drugs being much stronger drivers of placental transfer [159]. However, and despite the above considerations, protein binding of labetalol is unaltered, whereas the unbound fraction of propranolol increases during pregnancy [160].

## CONCLUSION AND FUTURE DIRECTIONS

Although adrenergic therapies are prescribed to improve maternal cardiovascular function during pregnancy, the data supporting the effect of these medications on fetal growth and development are lacking. All known adrenergic receptor subtypes are functionally expressed in the umbilico-fetoplacental vasculature. However, there remains little evidence for specific receptor expression in the microcirculation of the placental villi, particularly within stem arterioles, which are likely the most important locus in the regulation of placental vascular tone. Delineating the downstream pathways in the microcirculation of the placenta could provide important knowledge for prediction of the net effects on the fetus. The evidence presented in this review highlights the unique nature of placental adrenergic pharmacology and the dearth in knowledge of effects of antihypertensive and cardiac therapeutics on fetal blood flow through this vital organ. Understanding the vasoreactivity of the fetoplacental vasculature in response to locus-specific adrenergic activation/deactivation, and subsequently the impact on blood flow, is a key priority in obstetric research.

## ACKNOWLEDGEMENTS

T.T. was supported by a British Heart Foundation Project Grant (PG/19/25/34301) to P.B. For the remaining authors, none were declared.

Illustrative figures were created using BioRender.com.

### Conflicts of interest

All other authors declare no conflict of interests.

## Supplementary Material

Supplemental Digital Content
