# Supplementary material for: Fetoplacental vascular effects of maternal adrenergic antihypertensive and cardioprotective medications in pregnancy
Source: J Hypertens. 2023 Sep 11;41(11):1675–87. doi: 10.1097/HJH.0000000000003532 (PMC10552840; doi:10.1097/HJH.0000000000003532)
Supplement: Supplemental Digital Content [file jhype-41-1675-s001.docx]

**Supplementary Material**

**Supplementary Table 1: Prediction of likely vasoactive effects of adrenergic medications on the fetoplacental vasculature.**

| **Adrenergic**  **medication** | | **Receptor**  **targeted** | **Evoked effect**  **on EC**^֍^ | **Evoked effect on VSMC**^֍^ | **Intrinsic response**  **IC/EC_50_** | **Fetal plasma**  **levels** | **Systemic plasma**  **levels** | **Predicted**  **vasoactivity of dose** |
| --- | --- | --- | --- | --- | --- | --- | --- | --- |
| α-AR  agonist | *Clonidine*  Half-life:  11 hr (1)  F:M ratio  1.0 (2) | α_2A_-AR > α_2C_-AR > α_2B_-AR (3) | α_2_- and  α_2A_-AR:  relaxation  α_2B_- and α_2C_-AR:  unknown | α_2_-, α_2B_-, α_2C_-AR:  constriction | ~10^-7^M  - relaxation in rat mesenteric artery (4) | 2.6 x 10^-9^M^§^  - 0.30 mg/day  (oral)  - HUV, HUA (2) | P  2.6 x 10^-9^M  - 0.30 mg/day  (oral) (2) | Unlikely |
|  | *Methyldopa*  Half-life:  ~ 2 hr (5)  F:M ratio  1.4 (based on max dose regimen) (6) | α_2_-AR | α_2_-AR:  relaxation | α_2_-AR:  constriction | N/A | 4.12 x 10^-6^M^§^  - 2 g/day  (route, N/A)  -umbilical cord (6) | P   - 1. x 10^-6^M   - 2 g/day  (route, N/A) (6) | No fetoplacental physiological data available to predict a likely effect |
| α-AR  antagonist | *Prazosin*  Half-life:  ~3 hr (7) | α_1A_-AR >  α_1B_-AR >  α_1D_-AR(8) | α_1A_-AR:  unknown  α_1B_ and  α_1D_-AR: constriction | α_1A_-AR:  relaxation  α_1B_-AR: constriction  α_1D_-AR: relaxation | 2x10^-10^M  - relaxation in dog aorta (9) |  | NP  11.9 x 10^-9^M  -10 mg/twice a day (7)  52.2 x 10^-9^M  -1mg (IV) (10) | Likely to relax  if fetoplacental circulation is equally sensitive as dog aorta |
| β-AR  antagonist | *Labetalol*  ISA  (partial  β_2_-AR agonist)  Half-life:  5.8 hr  F:M ratio  0.44 (11) | β-AR >  α_1_-AR (12) | α_1_-AR:  constriction  β_1_-AR:  unknown  β_2_-AR:  constriction | α_1_-AR:  relaxation  β_2_-AR:  constriction  partial  β_2_-AR agonist:  relaxation | 10^-6^M  -constriction in human placental cotyledon (13) | - 1. x 10^-7^M^#^   -450mg/day  (oral) (11)  1.3 x 10^-7^M^#^  -330mg/day  (oral)  7 x 10^-8^M^#^  -400mg/day  (oral)  1.8 x 10^-7^M^#^  -600mg/day  (oral)  2.1 x 10^-7^M^#^  -700mg/day  (oral) (14) | P  3.4 x 10^-7^M^#^  -450 mg/day (11)  NP  1.74 x10^-5^M^§^  -120mg (IV)  1.75 x10^-6^M^§^  -120mg (IV, after 8.5 min) (15) | Unlikely at oral doses  Likely  constriction at IV doses |
|  | *Propanolol*  Half-life:  3-6 hr  F:M ratio^#^  0.32 (16) | β_1_- and  β_2_-AR >  β_3_-AR | β_1_-AR:  unknown  β_2_-AR:  constriction  β_3_-AR:  constriction | β_1_-AR:  constriction  β_2_-AR:  constriction  β_3_-AR:  constriction | 10^-6^M  ~ 16% relaxation of HPA, HPV (17)  10^-4^M  ~ 50% relaxation of human radial artery (18) | 5.4 x 10^-8^M^#^  -20mg/twice a day (oral) (19)  6.6 x 10^-8^M^#^  -80-240 mg/day (16) | P  7.3 x 10^-8^M^#^  -20mg/twice a day (oral) (19)  28.7 x10^-8^M^#^  -80-240 mg/day (16) | Unlikely |
|  | *Oxprenolol*  β-AR antagonist  partial  β-AR agonist (20)  Half-life:  1-3 hr  F:M ratio:  0.37 (21) | No-selectivity for  β-AR (20) | β_1_-AR:  unknown  β_2_-AR:  constriction  β_3_-AR:  constriction  ISA:  β-AR:  relaxation | β_1_-AR:  constriction  β_2_-AR:  constriction  β_3_-AR:  constriction  ISA:  β-AR:  relaxation | N/A | 7.1 x10^-8^M^#^  in HUA  8.1 x10^-8^M^#^  In HUV  -80 mg/three times daily  (oral) (21) | P  6.31 x10^-7^M^#^  -80 mg/ three times daily  (oral) (21) | No fetoplacental physiological data available to predict a likely effect |
|  | *Atenolol*  Half-life:  8.1 hr  F:M ratio:  0.9 (22) | β_1_-AR>  β_2_-AR(23) | β_1_-AR:  unknown  β_2_-AR:  constriction | β_1_-AR:  constriction  β_2_-AR:  constriction | N/A | 8.3 x10^-7^M^#^  -100 mg/daily  (oral) (22) | P  2.2 x10^-6^M^§^  -100 mg/daily  (oral) (22) | No fetoplacental physiological data available to predict a likely effect |
|  | *Bisoprolol*  Half-life:  10-12 hr (24) | β_1_-AR>  β_2_-AR (25) | β_1_-AR:  unknown  β_2_-AR:  constriction | β_1_-AR:  constriction  β_2_-AR:  constriction | N/A | N/A | NP  3.1x10^-9^M - 9x10^-7^M  1.25-10 mg/daily  (oral) (26) | No fetoplacental physiological data available to predict a likely effect |
|  | *Metoprolol*  Half-life:  3-4 hr  F:M ratio:  0.88-1.27 (27) | β_1_-AR>  β_2_-AR (23) | β_1_-AR:  unknown  β_2_-AR:  constriction | β_1_-AR:  constriction  β_2_-AR:  constriction |  | 4x10^-8^M^#^ (HUV)  4.3x10^-8^M^#^ (HUA)  -25-750 mg/daily (oral) (28) | P  7.8x10^-8^M^#^  -25-750 mg/daily (oral) (28) | No fetoplacental physiological data available to predict a likely effect |
|  | *Nebivolol*  Half-life:  10.3-31.9 hr (29) | β_1_-AR>  β_2_-AR (25)  β_3_-AR  agonist (30) | β_1_-AR:  unknown  β_2_-AR:  constriction  β_3_-AR:  relaxation (NO production) (31) | β_1_-AR:  constriction  β_2_-AR:  constriction | 10^-4^M  ~ 70% relaxation of human radial artery (18) | N/A | NP  4.1x10^-9^M^§^ (Extensive Metabolisers)  1.3x10^-8^M^§^  (Poor Metabolisers)  -5mg/daily  (oral) (32) | Unlikely |
|  | *Celiprolol*  ISA  (partial  β_2_-AR agonist) (33)  Half-life:  4-5 hr  F:M ratio:  0.25-0.50 (34) | β_1_-AR>  α_1_-AR (35) | β_1_-AR:  unknown  α_1_-AR:  constriction  ISA  β_2_-AR:  relaxation | β_1_-AR:  constriction  α_1_-AR:  relaxation  ISA  β_2_-AR:  relaxation | 10^-4^M  ~ 14% relaxation of guinea pig mesenteric artery (33) | N/A | NP  6.8x10^-9^M^§^  -600mg/daily  (oral) (36) | Unlikely |

Plasma levels: ^#^ mean, ^§^maximum. HUV: Human Umbilical Vein. HUA: Human Umbilical Artery. HPA: Human Placental Artery. HPV: Human Placental Vein. ISA: Intrinsic Sympathomimetic Activity. P: Pregnant. NP: Non-Pregnant. IV: Intravenous Injection. ^֍^: evoked effect is based on table 3 in the main text. NO: nitric oxide. IC50: half-maximal inhibitory concentration. EC50: half-maximal effective concentration.

**Supplementary References**

1. Cunningham FE, Baughman VL, Peters J, Laurito CE. Comparative pharmacokinetics of oral versus sublingual clonidine. J Clin Anesth. 1994;6(5):430-3.

2. Buchanan ML, Easterling TR, Carr DB, Shen DD, Risler LJ, Nelson WL, et al. Clonidine pharmacokinetics in pregnancy. Drug Metab Dispos. 2009;37(4):702-5.

3. Jasper JR, Lesnick JD, Chang LK, Yamanishi SS, Chang TK, Hsu SA, et al. Ligand efficacy and potency at recombinant alpha2 adrenergic receptors: agonist-mediated [35S]GTPgammaS binding. Biochem Pharmacol. 1998;55(7):1035-43.

4. Figueroa XF, Poblete MI, Boric MP, Mendizábal VE, Adler-Graschinsky E, Huidobro-Toro JP. Clonidine-induced nitric oxide-dependent vasorelaxation mediated by endothelial alpha(2)-adrenoceptor activation. Br J Pharmacol. 2001;134(5):957-68.

5. Barnett AJ, Bobik A, Carson V, Korman JS, McLean AJ. Pharmacokinetics of methyldopa. Plasma levels following single intravenous, oral and multiple oral dosage in normotensive and hypertensive subjects. Clin Exp Pharmacol Physiol. 1977;4(4):331-9.

6. Jones HM, Cummings AJ, Setchell KD, Lawson AM. A study of the disposition of alpha-methyldopa in newborn infants following its administration to the mother for the treatment of hypertension during pregnancy. Br J Clin Pharmacol. 1979;8(5):433-40.

7. Larochelle P, du Souich P, Hamet P, Larocque P, Armstrong J. Prazosin plasma concentration and blood pressure reduction. Hypertension. 1982;4(1):93-101.

8. Proudman RGW, Pupo AS, Baker JG. The affinity and selectivity of α-adrenoceptor antagonists, antidepressants, and antipsychotics for the human α1A, α1B, and α1D-adrenoceptors. Pharmacol Res Perspect. 2020;8(4):e00602.

9. Nagatoma T, Tsuchihashi H, Sasaki S, Nakagawa Y, Nakahara H, Imai S. Displacement by alpha-adrenergic agonists and antagonists of 3H-prazosin bound to the alpha-adrenoceptors of the dog aorta and the rat brain. Jpn J Pharmacol. 1985;37(2):181-7.

10. Bateman DN, Hobbs DC, Twomey TM, Stevens EA, Rawlins MD. Prazosin, pharmacokinetics and concentration effect. Eur J Clin Pharmacol. 1979;16(3):177-81.

11. Saotome T, Minoura S, Terashi K, Sato T, Echizen H, Ishizaki T. Labetalol in hypertension during the third trimester of pregnancy: its antihypertensive effect and pharmacokinetic-dynamic analysis. J Clin Pharmacol. 1993;33(10):979-88.

12. Aggerbeck M, Guellaen G, Hanoune J. Biochemical evidence for the dual action of labetalol on alpha- and beta-adrenoceptors. Br J Pharmacol. 1978;62(4):543-8.

13. Petersen OB, Skajaa K, Svane D, Gregersen H, Forman A. The effects of dihydralazine, labetalol and magnesium sulphate on the isolated, perfused human placental cotyledon. Br J Obstet Gynaecol. 1994;101(10):871-8.

14. Michael CA. Use of labetalol in the treatment of severe hypertension during pregnancy. Br J Clin Pharmacol. 1979;8(Suppl 2):211s-5s.

15. Richards DA. Pharmacological effects of labetalol in man. Br J Clin Pharmacol. 1976;3(4 Suppl 3):721-3.

16. Smith MT, Livingstone I, Eadie MJ, Hooper WD, Triggs EJ. Metabolism of propranolol in the human maternal-placental-foetal unit. Eur J Clin Pharmacol. 1983;24(6):727-32.

17. Omar HA, Rhodes LA, Ramirez R, Arsich J, Einzig S. Alteration of human placental vascular tone by antiarrhythmic medications in vitro. J Cardiovasc Electrophysiol. 1996;7(12):1197-203.

18. Korkmaz O, Saraç B, Göksel S, Yildirim S, Berkan O, Bagcivan I. Labetalol, nebivolol, and propranolol relax human radial artery used as coronary bypass graft. J Thorac Cardiovasc Surg. 2015;149(4):1036-40.

19. Taylor EA, Turner P. Anti-hypertensive therapy with propranolol during pregnancy and lactation. Postgrad Med J. 1981;57(669):427-30.

20. Kendall MJ, John VA. Oxprenolol: clinical pharmacology, pharmacokinetics, and pharmacodynamics. Am J Cardiol. 1983;52(9):27d-33d.

21. Sioufi A, Hillion D, Lumbroso P, Wainer R, Olivier-Martin M, Schoeller JP, et al. Oxprenolol placental transfer, plasma concentrations in newborns and passage into breast milk. Br J Clin Pharmacol. 1984;18(3):453-6.

22. Thorley KJ, McAinsh J, Cruickshank JM. Atenolol in the treatment of pregnancy-induced hypertension. Br J Clin Pharmacol. 1981;12(5):725-30.

23. Abrahamsson T, Ek B, Nerme V. The beta 1- and beta 2-adrenoceptor affinity of atenolol and metoprolol. A receptor-binding study performed with different radioligands in tissues from the rat, the guinea pig and man. Biochem Pharmacol. 1988;37(2):203-8.

24. Leopold G. Balanced pharmacokinetics and metabolism of bisoprolol. J Cardiovasc Pharmacol. 1986;8 Suppl 11:S16-20.

25. Bristow MR. beta-adrenergic receptor blockade in chronic heart failure. Circulation. 2000;101(5):558-69.

26. Nikolic VN, Jankovic SM, Dimitrijevic ZM, Sokolovic MJ, Andric BR, Petrovic DS, et al. Population Pharmacokinetics of Bisoprolol in Hemodialysis Patients with Hypertension. Pharmacology. 2016;97(3-4):134-7.

27. Mitani GM, Steinberg I, Lien EJ, Harrison EC, Elkayam U. The pharmacokinetics of antiarrhythmic agents in pregnancy and lactation. Clin Pharmacokinet. 1987;12(4):253-91.

28. Ryu RJ, Eyal S, Easterling TR, Caritis SN, Venkataraman R, Hankins G, et al. Pharmacokinetics of metoprolol during pregnancy and lactation. J Clin Pharmacol. 2016;56(5):581-9.

29. Hilas O, Ezzo D. Nebivolol (bystolic), a novel Beta blocker for hypertension. P T. 2009;34(4):188-92.

30. Gosgnach W, Boixel C, Névo N, Poiraud T, Michel JB. Nebivolol induces calcium-independent signaling in endothelial cells by a possible beta-adrenergic pathway. J Cardiovasc Pharmacol. 2001;38(2):191-9.

31. Howlett JG. Nebivolol: vasodilator properties and evidence for relevance in treatment of cardiovascular disease. Can J Cardiol. 2014;30(5 Suppl):S29-37.

32. Briciu C, Neag M, Muntean D, Bocsan C, Buzoianu A, Antonescu O, et al. Phenotypic differences in nebivolol metabolism and bioavailability in healthy volunteers. Clujul Med. 2015;88(2):208-13.

33. Dhein S, Titzer S, Wallstein M, Müller A, Gerwin R, Panzner B, et al. Celiprolol exerts microvascular dilatation by activation of beta 2-adrenoceptors. Naunyn Schmiedebergs Arch Pharmacol. 1992;346(1):27-31.

34. Kofahl B, Henke D, Hettenbach A, Mutschler E. Studies on placental transfer of celiprolol. Eur J Clin Pharmacol. 1993;44(4):381-2.

35. Monopoli A, Forlani A, Bevilacqua M, Vago T, Norbiato G, Bertora P, et al. Interaction of selected vasodilating beta-blockers with adrenergic receptors in human cardiovascular tissues. J Cardiovasc Pharmacol. 1989;14(1):114-20.

36. Norris RJ, Lee EH, Muirhead D, Sanders SW. A pharmacokinetic evaluation of celiprolol in healthy elderly volunteers. J Cardiovasc Pharmacol. 1986;8 Suppl 4:S91-2.
